# A novel prognostic signature based on cuproptosis-related lncRNA mining in colorectal cancer

**DOI:** 10.3389/fgene.2022.969845

**Published:** 2022-08-29

**Authors:** Dong Hou, Jia-nan Tan, Sheng-ning Zhou, Xu Yang, Zhi-hong Zhang, Guang-yu Zhong, Lin Zhong, Bin Yang, Fang-hai Han

**Affiliations:** Department of Gastrointestinal Surgery, Sun Yat-Sen Memorial Hospital, Sun Yat-Sen University, Guangzhou, China

**Keywords:** cuproptosis, lncRNA, colorectal cancer, consistent clustering, immune microenvironment, tumor mutational burden (TMB)

## Abstract

**Background:** Colorectal cancer (CRC) is a common malignant tumor that affects the large bowel or the rectum. Cuproptosis, recently discovered programmed cell death process, may play an important role in CRC tumorigenesis. Long non-coding RNAs (lncRNAs) can alter the proliferation of colorectal cancer cells through the control and activation of gene expression. To date, cuproptosis-related lncRNAs, have not been investigated as potential predictive biomarkers in colorectal cancer.

**Methods:** The mRNA and lncRNA expression data of colorectal cancer were gathered from The Tumor Genome Atlas (TCGA) database, and Pearson correlation analysis and univariate Cox regression analysis were used to identify the lncRNAs with differential prognosis. Colorectal cancer was classified using consistent clustering, and the clinical significance of different types, tumor heterogeneity, and immune microenvironment differences was investigated. The differential lncRNAs were further screened using LASSO regression to develop a risk scoring model, which was then paired with clinicopathological variables to create a nomogram. Finally, the copy number changes in the high-risk and low-risk groups were compared.

**Results:** Two clusters were formed based on the 28 prognostic cuproptosis-related lncRNAs, and the prognosis of cluster 2 was found to be significantly lower than that of cluster 1. Cluster 1 showed increased immune cell infiltration and immunological score, as well as strong enrichment of immune checkpoint genes. Next, LASSO regression was used to select 11 distinctive lncRNAs, and a risk score model was constructed using the training set to distinguish between high and low-risk groups. Patients in the high-risk group had a lower survival rate than those in the low-risk group, and both the test set and the total set produced consistent results. The AUC value of the ROC curve revealed the scoring model’s efficacy in predicting long-term OS in patients. Moreover, the model could be used as an independent predictor when combined with a multivariate analysis of clinicopathological features, and our nomogram could be used intuitively to predict prognosis.

**Conclusion:** Collectively, we developed a risk model using 11 differential lncRNAs and demonstrated that the model has predictive value as well as clinical and therapeutic implications for predicting prognosis in CRC patients.

## Introduction

Colorectal cancer (CRC) is an exceptionally frequent illness worldwide. There are roughly 408,000 new cases with colorectal cancer and 196,000 deaths per year, ranking second in terms of incidence and fourth in terms of mortality (Sung et al., 2021; Zheng, 2022). Since the early clinical indications of colorectal cancer are not apparent, the cancer is often medium and advanced stages detection. Radical removal of primary tumor and lymph node dissection are the major therapeutic strategies for colorectal malignancies. The introduction of Total Mesorectal Excision (TME) and Complete Mesocolic Excision (CME) (Heald et al., 1982; Hohenberger et al., 2009) has reduced the local recurrence rate from the original 20–40 percent to 3-8 percent, thus dramatically improving the overall survival rate of patients, which can be described as a milestone in colorectal surgery (Carlsen et al., 1998; Dattani et al., 2016). However, the prognosis of minority are still poor, massive investigations revealed that this may be connected with Copy Number Variation (CNV), tumor microenvironment and tumor heterogeneity (Sagaert et al., 2018).

Tsvetkov et al. demonstrated in human cells that copper-dependent, controlled cell death is separate from known death mechanisms and is dependent on mitochondrial respiration, which is dubbed cupropotosis. The work has proven that cupropotosis occurs by means of direct binding of copper to lipoylated components of the tricarboxylic acid (TCA) cycle (Tsvetkov et al., 2022). This results in lipoylated protein aggregation and subsequent iron-sulfur cluster protein loss, which leads to proteotoxic stress and ultimately cell death. Cupropotosis may explain the pathology associated with genetic copper overload disorders and suggest new ways to harness copper toxicity to treat cancer, which may be especially useful for cancers that are naturally resistant to apoptosis, enabling a new way to kill cancer cells by exploiting the distinct action of this metal (Kahlson and Dixon, 2022; Oliveri, 2022).

In animals, long no-coding RNAs (lncRNAs) affect roughly 70 percent genes expression by interacting with DNA replication, RNA transcription, protein translation. Increasing research suggested that abnormal expression of lncRNA impairs homeostasis in organisms and may promote or inhibit some cancers (Li et al., 2018; Chen et al., 2021). The lncRNA NEAT1 has been demonstrated to stimulate Wnt/β-catenin signaling and increases colorectal cancer progression *via* combining with DDX5. Another study demonstrated that circulating lncRNA SNHG11 as a new biomarker for early diagnosis and prognosis of colorectal cancer1 (Xu et al., 2020). However, the function of cupropotosis-related lncRNAs is uncertain in colorectal cancer. Therefore, it is crucial to research cupropotosis-related lncRNAs and to screen predictive biomarkers among these lncRNAs.

We found predictive biomarkers based on cupropotosis related-lncRNA and created a prognosis model for colorectal cancer by using the lncRNA expression patterns of The Cancer Genome Atlas (TCGA) colorectal cancer cohort. We also study the link between the signature lncRNA and tumor mutational burden (TMB) in colorectal cancer.

## Materials and methods

### Data gathering and preparation

The TCGA database (https://cancergenome.nih.gov/) was used to gather RNA transcriptome analysis data as well as patient clinical information. The Fragment Per Kilobase Method (FPKM) was used to gather transcriptome data, and clinical information included age, sex, grade, and survival status. After screening for patients with a survival duration of more than 1 day, 487 patients were eventually included in the study, with 446 tumor samples and 41 normal samples. We found 19 genes linked to cuprpptosis, including NFE2L2, NLRP3, ATP7B, ATP7A, SLC31A1, FDX1, LIAS, LIPT1, LIPT2, DLD, DLAT, PDHA1, PDHB, MTF1, GLS, CDKN2A, DBT, GCSH, DLST, according to previously published research (Tsvetkov et al., 2022). We obtained 16,773 lncRNAs through the annotation of integrated IDs in the TCGA dataset, which we obtained from the GENCODE website (https://www.gencodegenes.org). The lncRNAs expression matrix was then extracted from the TCGA dataset using Perl. Pearson correlation analysis was carried out between these lncRNAs and 19 copper death-related genes, with Pearson R > 0, 5 and a *p* value of 0.001. A total of 1,305 lncRNAs related to cuproptosis were discovered. Finally, to facilitate further analysis, we combined the expression data of these lncRNAs with the survival time and survival status of patients in clinical data.

### Identify prognosis-related lncRNAs and perform cluster analysis on the samples

The “survival” package in R software was used to perform univariate cox regression analysis on all cuproptosis-related lncRNAs, with *p* < 0.05 set as the screening condition, and 28 lncRNAs with significant differences in tumor prognosis were obtained and plotted based on the regression analysis results. The forest map is graphically shown, and the “pheatmap” software program builds a heat map to show the expression of prognosis-related lncRNAs in CRC and normal tissues. The prognostic-related lncRNA expression data was then grouped using the “ConsensusClusterPlus” software tool to classify colorectal cancer into distinct subtypes. The “pheatmap” software was used to visualize gene expression in different subtypes. Kaplan–Meier survival curves were used to compare survival outcomes between subgroups.

### Tumor immune micro-environment analysis based on cluster typing

To examine the immunological milieu of colorectal cancer tissue in samples based on CIBERSORT (Newman et al., 2015), we quantified 22 immune cells in CRC samples, including B cells, The infiltrating abundance of T cells, natural killer cells, macrophages, dendritic cells, eosinophils, and neutrophils was compared between different subtypes using the “vioplot” and “ggpubr” packages in the R software. Abundant expression of immune-infiltrating cells was visualized. Then, ESTIMATE, a method that employs expression data to estimate stromal and immune cell scores in malignant tumor tissue, was utilized to predict tumor purity in the TME (Yoshihara et al., 2013). collected the immuneScore, stromalScore and ESTIMATEScore scores of colorectal cancer tissues, and did differential analysis between distinct subtypes of tissues with the “limma” software package.

### Construction and evaluation of a risk model for copper death-related lncRNA prediction

To create an efficient prognostic risk model, we randomly separated colon cancer patients into training and test sets using the “caret” program. This training set is used to develop prognostic features and evaluate them in the test set. The Least Absolute Shrinkage and Selection Operator (LASSO) regression analysis was performed to further screen the prognosis-related lncRNAs to minimize overfitting. Subsequently, multivariate Cox proportional hazards regression analysis was performed to uncover independent prognostic markers, while the c-index was generated using the ‘survminer’ software package to estimate the model’s best prediction. Ultimately, 11 lncRNAs linked with prognosis risk were found. The risk score formula is given as follows: Risk Score = Coe1∗Exp1+Coe2∗Exp2+Coe3∗Exp3+…+Coen∗Expn. Coe is the coefficient of multiple Cox regression analysis of 11 lncRNAs, and Exp is the associated expression value. Colorectal cancer patients were split into high-risk and low-risk groups according to the median risk score. The survival outcomes of the two groups were compared using Kaplan–Meier survival curves. Supplementary files for survival information were downloaded from the Xena database (http://xena.ucsc.edu/), and the “survival” software tool was used to evaluate disease-free progression (PFS) between the two groups. Receiver operating characteristic (ROC) curves were constructed using the “survival” and “timeROC” programs in the R software. These ROC curves were used to study the 1-, 3-, and 5-year survival rates of patients, utilizing the receiver operating characteristic curve (ROC) and its area under the curve (AUC) value to measure the specificity and sensitivity of the signature signal.

### Construction of nomogram and principal component analysis

By combining independent factors found by multivariate Cox regression analysis, a nomogram model was created to examine the prediction ability of the predictive model for 1-, 3-, and 5-year OS rates. And a calibration plot was constructed to produce a concordance index to assess the accuracy of the nomogram’s prediction power. Through PCA analysis and hierarchical clustering of all samples, the distribution of all samples was shown by 3D scatter plot.

### Gene set enrichment analysis

In order to explore the potential biological processes and cellular pathways related to the expression of lncRNAs related to cuproptosis, input files were prepared according to the gene expression profiles of patients in high and low risk groups obtained by risk scoring, and GSEA software was used to conduct GSEA to study enrichment projects. Simulated value = 1,000, *p* value <0.05 and was picked as the criteria for statistical significance.

### Assessment of tumor mutational load and immune function analyses based on the copper death-related lncRNA prediction risk model

Simple nucleotide variation data relating to CRC patients were collected from the TCGA database (https://cancergenome.nih.gov//). Then, the samples were divided into high- and low-risk groups according to the prediction model risk score using Perl language, and the differences in tumor mutational burden (TMB) and somatic mutations in the samples from the high-risk group and the low-risk group were compared using “maftools” in R. According to TMB levels, patients were separated into high and low TMB groups, and Kaplan-Meier survival analysis was done. The “limma, pheatmap, ggpubr, reshape2″ software programs were used to construct boxplots and heat maps to demonstrate the variations in immune cell infiltration levels and immune function between high and low risk groups, respectively. Finally, according to the TIDE of colorectal cancer samples in the TCGA database retrieved from the online domain (http://tide.dfci.harvard.edu/login/), the “limma, ggpubr” software package was used to map the link between high and low risk groups. TIDE compares violin graphs.

### Statistical analysis

All statistical analyses in this work were done with software R x64 4.1.3, and statistical significance was fixed at *p* < 0.05. OS rates were expressed using Kaplan-Meier curves and log-rank tests were used to determine statistically significant differences. Univariate and multivariate Cox regression analyses were utilized to validate risk scores of signature lncRNAs as independent predictive variables. The “timeROC” software package was used to generate the ROC curve and determine the area under the curve (AUC) (AUC). The “GSVA, GSEABase” software suite was utilized to undertake immune infiltration analyses.

## Results

### Cuproptosis-related lncRNAs and their prognostic value

**FIGURE 1:**
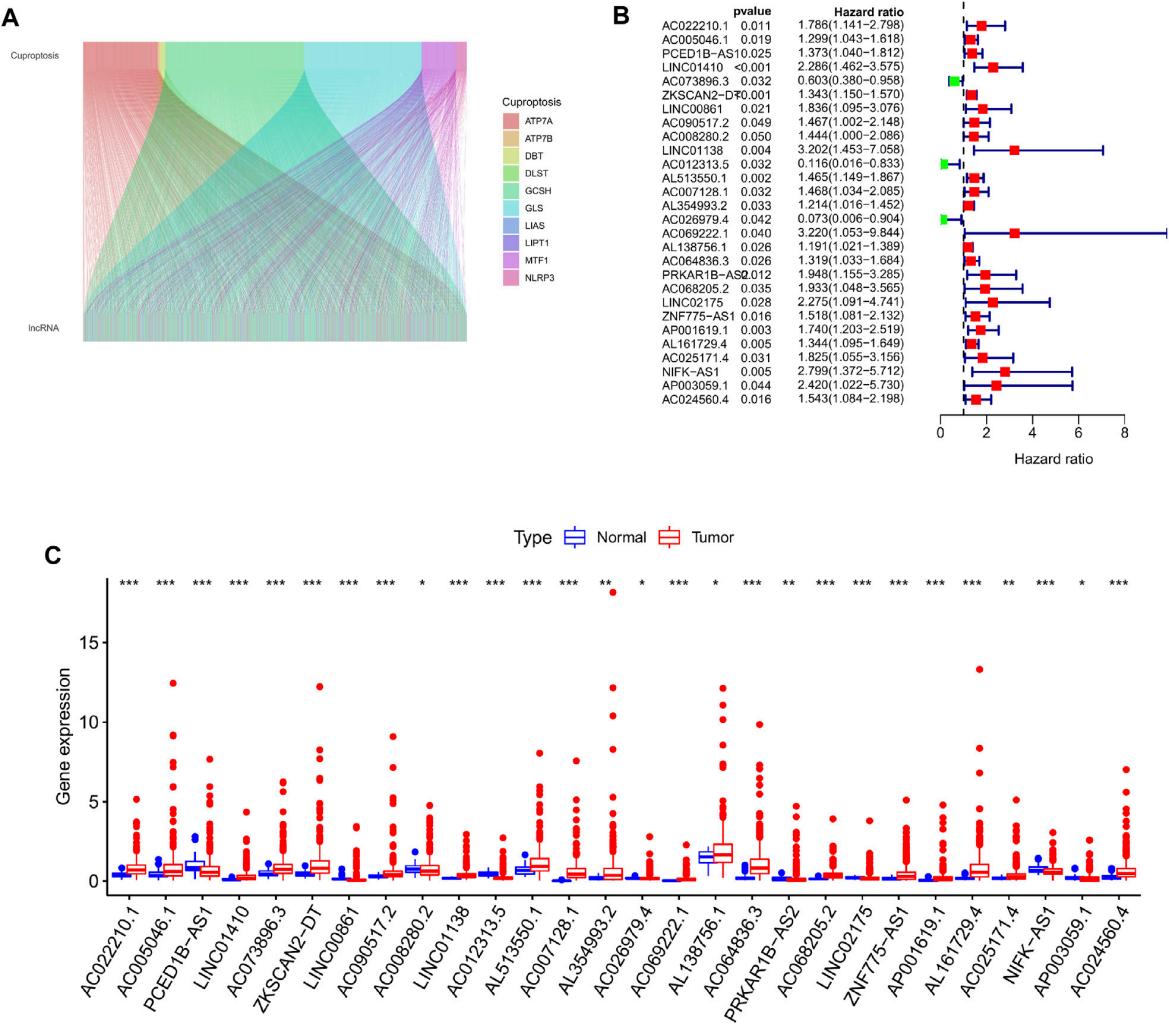
Screening for cuproptosis-related lncRNAs with prognostic differences. **(A)** Mulberry plot showing the co-expression of cuproptosis-related genes and lncRNAs. **(B)** 28 prognostic differential lncRNA forest maps were drawn based on the univariate Cox regression results. **(C)** Boxplot showing the differential expression of 28 lncRNAs with prognostic differences in tumor tissue and normal tissue.

The RNA transcriptome data of 446 colorectal cancer patient samples acquired from the TCGA database were processed and sorted, and 16,773 lncRNAs were obtained through annotation. Then, Pearson correlation analysis was used to examine the link between 19 Cuproptosis mechanism-related genes and the acquired lncRNAs, and a total of 1,305 Cuproptosis mechanism-related lncRNAs were screened. (Figure 1A), subsequently, by combining clinical information (survival time and survival status of patients), we ran univariate cox regression analysis on these 1,305 lncRNAs one by one, with *p* < 0.05 as the screening criteria, a total of 28 prognostic variables were found. Finally, comparing the expression levels of these 28 lncRNAs in colorectal cancer tissues and normal tissues, we observed significant differences in the expression of these lncRNAs, among which 20 lncRNAs were up-regulated, including: AC022210.1 AC005046.1 LINC01410 AC073896.3 ZKSCAN2-DT AC090517.2 LINC01138 AL513550.1 AC007128.1 AL354993.2 AC069222.1 AL138756.1 AC064836.3 AC068205.2 ZNF775-AS1 AP001619.1 AL161729.4 AC0251.02456 AP0. Eight lncRNAs were down-regulated, including: PCED1B-AS1 LINC00861 AC008280.2 AC012313.5 AC026979.4 PRKAR1B-AS2 LINC02175 NIFK-AS1 (Figures 1B,C).

### Consistent clustering analysis based on the lncRNA linked with the prognosis of cuproptosis

**FIGURE 2:**
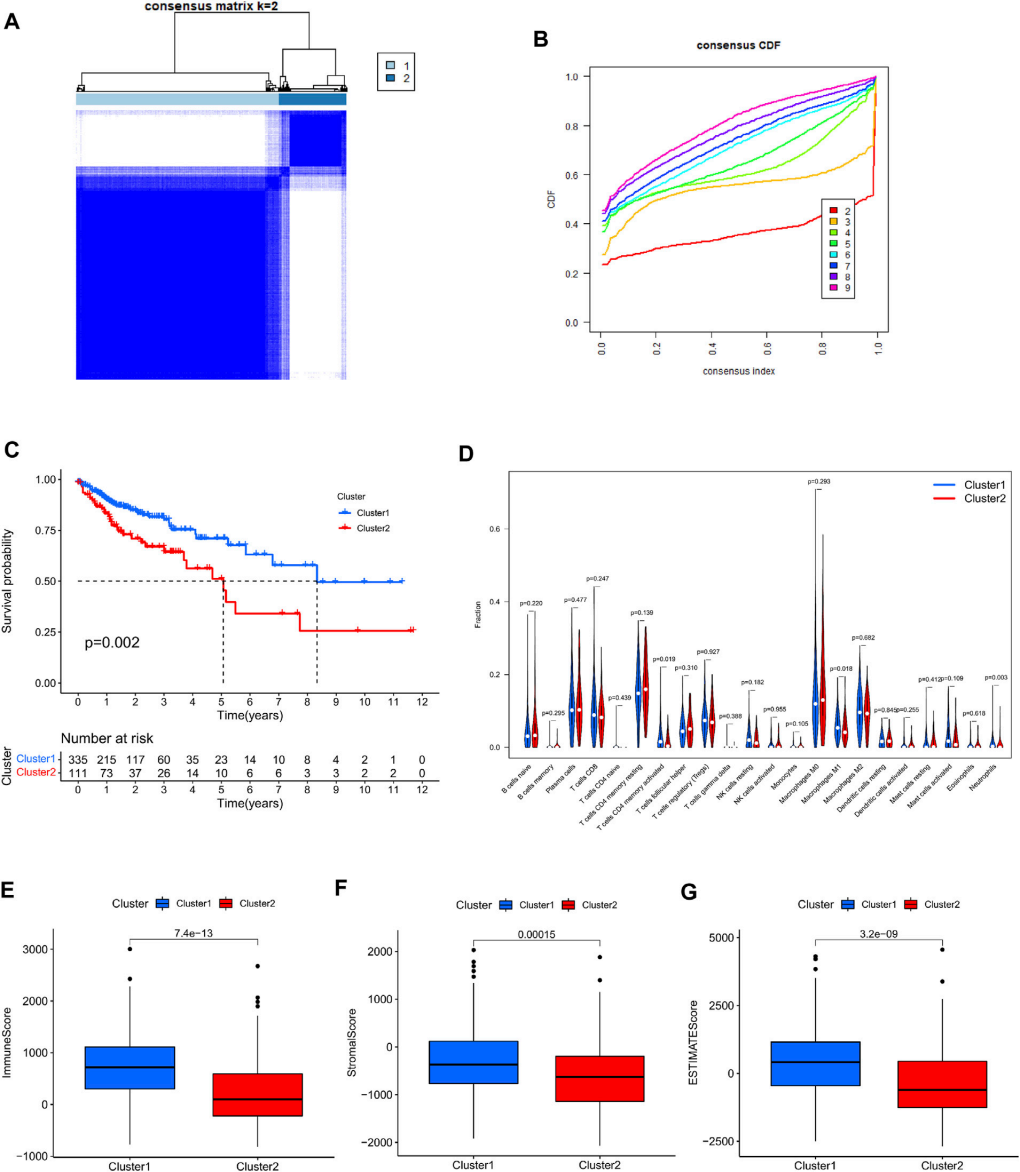
Consensus clustering analysis based on cuproptosis prognosis-associated lncRNAs. **(A)** 446 colon cancer patients were divided into two clusters (*k* = 2) according to a consistent clustering matrix. **(B)** Relative change in area under the CDF curve for *k* = 2–9. **(C)** Kaplan-Meier curve of overall survival in colon cancer patients with two clusters. **(D)** Infiltration of 22 immune-infiltrating cells in clusters 1 and 2 Abundance. **(E–G)** Comparison of immune scores, stromal scores, and assessment scores for cluster 1 and cluster 2.

Using a consistent clustering method to group 446 colorectal cancer patients according to the expression of cuproptosis prognosis-related lncRNAs in the dataset, we discovered that when the patients were separated into two subgroups, each group exhibited the best clustering stability (Figure 2A). Cumulative distribution functions, AUC increments when pooled are displayed (Figure 2B). Ultimately, we grouped all patients into cluster 1 (335 cases) and cluster 2 (111 instances) (111 cases). Then, we compared the survival data between different clusters by creating the Kaplan–Meier survival curve. It can be seen that the overall survival rate of cluster 2 was lower than that of cluster 1, and the difference was significant *p* = 0.002 (Figure 2C). Then we sought to analyze the tumor immune microenvironment between different groups. First, we assessed the infiltration abundance of 22 types of immune cells based on CIBERSORT, and performed differential analysis between clusters, the infiltration abundance of T cells CD4 memory activated, Macrophages M1, and Neutrophils was higher in cluster 1 (Figure 2D). The immune Score, stromal Score, ESTIMATE Score in colorectal cancer tissues from all patients in the sample were then assessed using the ESTIMATE method, and scores were compared between the different clusters. We discovered that cluster 1 all scored higher than cluster 2 with a significant difference (Figures 2E–G).

**FIGURE 3:**
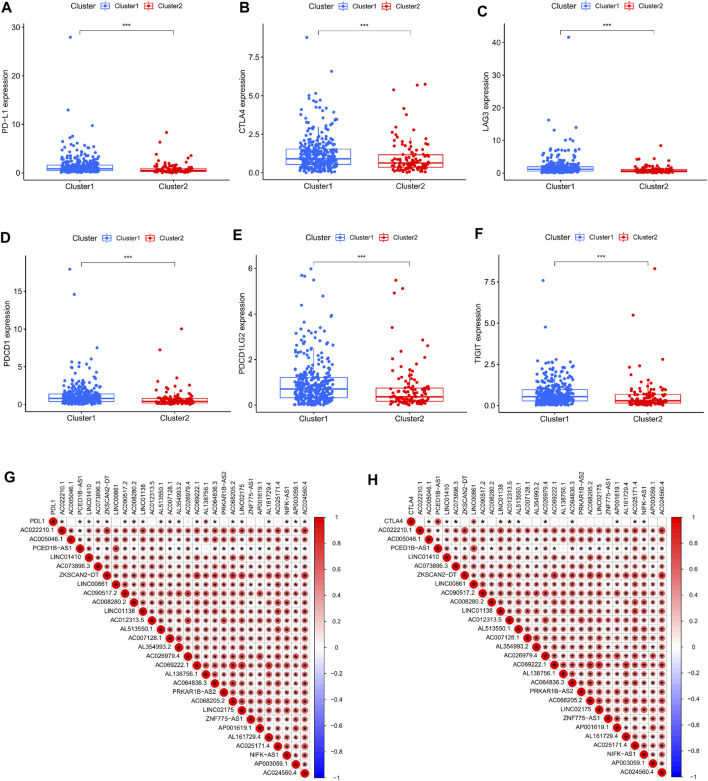
Correlation between immune checkpoints and cluster typing. **(A–F)** Expression levels of PD-L1, CTLA4, LAG3, PDCD1, PDCD1LG2, TIGIT in clusters 1 and 2. **(G–H)** Correlation of PD-L1, CTLA4 and cuproptosis-related lncRNAs.

At the same time, in order to investigate the relationship between type and immune checkpoints, we selected genes related to tumor immune checkpoints: PD-L1, CTLA4, LAG3, PDCD1, PDCD1LG2, and TIGIT. Through differential analysis and comparison, it can be observed that the expression of these genes in cluster 1 is dramatically elevated (Figures 3A–F). Correlation analysis between immune checkpoint genes and prognosis-related lncRNAs, we observed: PD-L1 expression was favorably connected with AL138756.1 (Figure 3G), CTLA4 expression was significantly correlated with Expression was positively linked with PCED1B−AS1, LINC00861 (Figure 3H). The expressions of LAG3,PDCD1, PDCD1LG2 and TIGIT were strongly linked with the expression of lncRNA:PCED1B-AS1 (Supplementary Figure S1).

### Construction of a cuproptosis-related lncRNA risk prediction model

**FIGURE 4:**
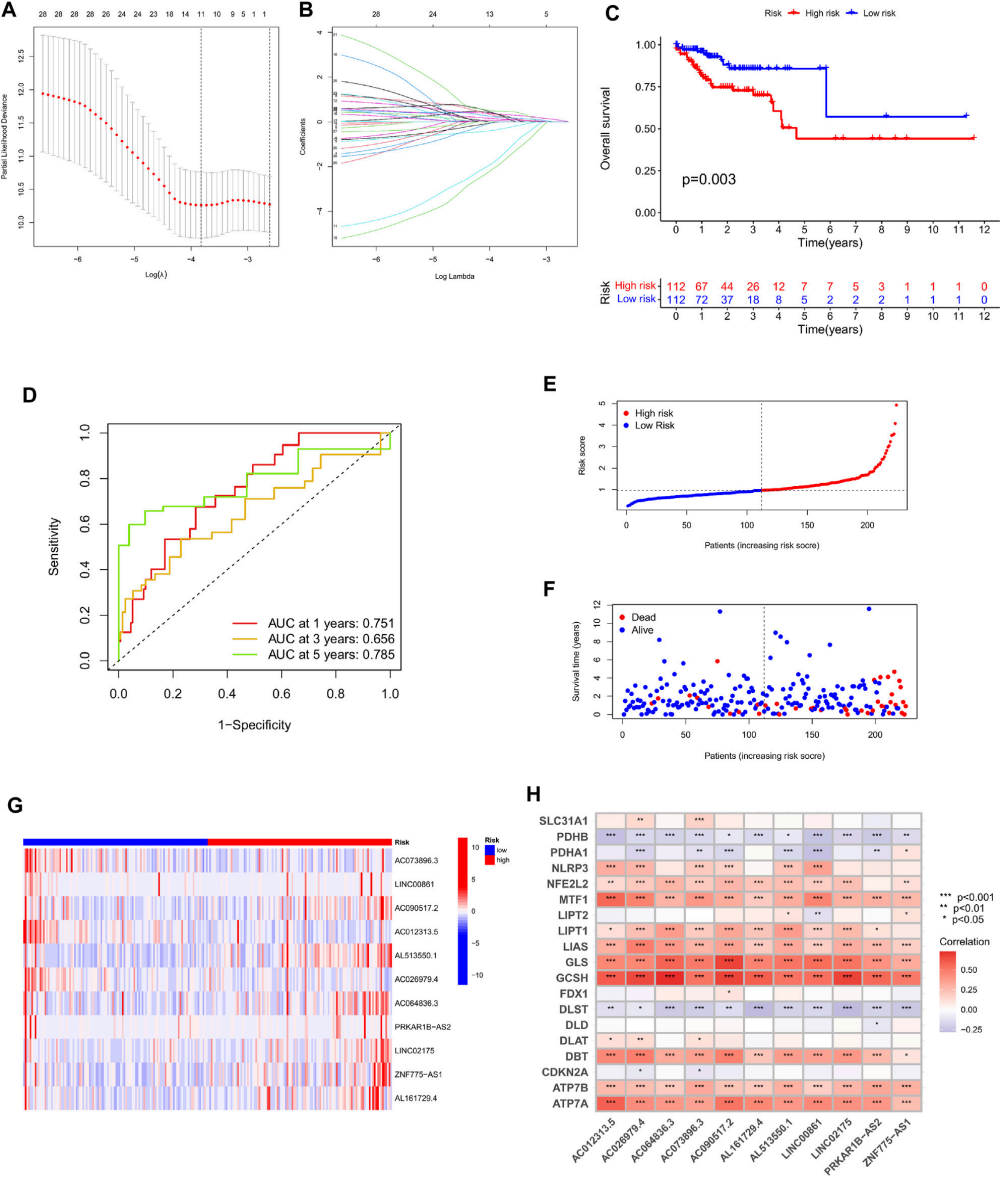
Construction and evaluation of OS-related cuproptosis-related lncRNA risk scoring models in the training cohort. **(A)** LASSO coefficient curves of 11 prognosis-related lncRNAs. **(B)** Ten-fold cross-validation of tuning parameter selection in the LASSO model. **(C)** Kaplan-Meier curves of survival outcomes for patients in the high and low risk groups. **(D)** 1-year, 3-year, and 5-year ROC curves and AUC values of the risk scoring model. **(E)** Risk score distribution. Blue dots represent risk scores for low-risk patients; red dots represent risk scores for high-risk patients. **(F)** Relationship between survival status and risk score. The horizontal axis represents the number of patients, and the vertical axis represents the risk score. Red dots represent dead patients and blue dots represent surviving patients. **(G)** Heatmap showing the expression profiles of 11 characteristic lncRNAs. **(H)** Heatmap of the association of 11 characteristic lncRNAs with cuproptosis-related genes.

In order to further examine the lncRNAs closely associated to the survival status of colorectal cancer patients, screen out possible prognostic indicators and validate the validity of the established prognostic risk model. First, we separated a total of 446 patients into a training set (224 cases) and a test set (222 cases) by random sampling. This training set was used to develop a prognostic risk model, which was tested in the test set. Second, we further performed LASSO regression analysis on the lncRNAs in the training set that were differentially related to the prognosis of colorectal cancer patients, selectively put lncRNAs into the model to obtain better performance parameters, and controlled the complexity of the model through a series of parameters to avoid overfitting. Finally, we integrated 11 lncRNAs into the construction of the model (Figures 4A,B). Multivariate COX regression analysis was done on these lncRNAs to derive their regression coefficients and consequently the risk scoring model for each patient in the sample: Exp AC073896.3x (-.326978990730302) + ExpLINC00861 × 0.324619815100717 + ExpAC090517.2 × 0.2 46047828207131 + ExpAC01233 .5 × (1.59222059316072) + ExpAL513550.1 × 0.344734659199596 + ExpAC026979.4x (1.88833971363086)+ExpAC064836.3 × 0.0750365708226491 + ExpPRKAR1BAS2x0.301585745373072 + ExpLINC02175 × 0.471828759105413 + ExpZNF775AS1x0.211601121547418 + ExpAL161729.4 × 0.131426560177917。 Afterwards, patients in the training set were split into high-risk and low-risk groups based on their median risk score. Kaplan-Meier survival curves showed that patients in the high-risk group had considerably lower overall survival than the low-risk group in the training set (Figure 4C). The prognosis survival rate of the patients was revealed by the ROC curve, and the AUC values at 1, 3, and 5 years were 0.751, 0.656, and 0.785, respectively (Figure 4D). Therefore, the time-dependent ROC curve validates the performance of the prediction model. In addition, by plotting the risk assessment scatterplot and heatmap of the training set, it can be seen that the survival time of patients in the high risk group was significantly lower (Figures 4E,F), and the expression of 11 prognosis-related lncRNAs had significant differences among different groups difference (Figure 4G). Finally, we ran a correlation study on the 11 lncRNAs included in the model and genes linked to Cuproptosis, and it was observed that the expression of various lncRNAs was strongly correlated with the expressions of MTF1, LIAS, GLS, GCSH, DBT, ATP7A, and ATP7B. Significant positive association, and negative correlation with the expression of PDHB, DLST (Figure 4H).

### Validation of predicted risk models

**FIGURE 5:**
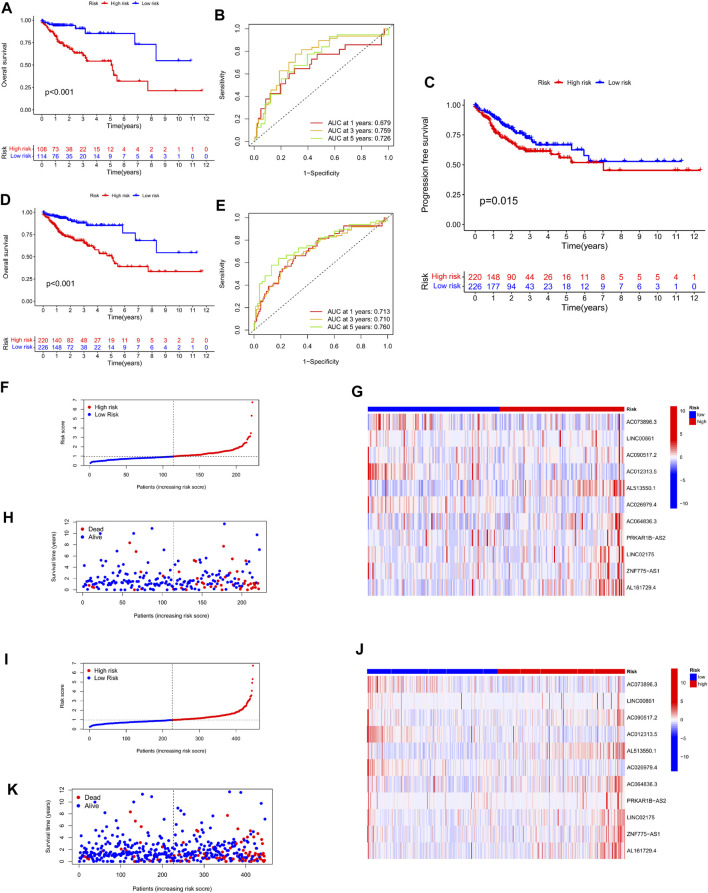
Validation of the stability of the risk scoring model in the test cohort and the total cohort. **(A,B)** Survival curves of high- and low-risk groups in the test cohort, and ROC curves and AUC values of the model at 1, 3, and 5 years. **(C)** Progression-free survival curves of patients in the high and low risk groups in the total cohort. **(D–E)** Survival curves of high- and low-risk groups in the total cohort, and ROC curves and AUC values of the model at 1, 3, and 5 years. **(F–H)** Scatter plots and heatmaps of expression levels of characteristic lncRNAs for patient risk score assessment in the test cohort. **(I–K)** Scatter plots and heatmaps of expression of characteristic lncRNAs for patient risk score assessment in the test cohort.

To demonstrate the robustness of the predictive risk model, we risk-scored 222 patients in the test and overall sets based on the same scoring model, and separated them into high- and low-risk groups. Similarly, in the Kaplan-Meier survival curve display, the survival rate of the high-risk group in both the test set and the total set was lower than that of the low-risk group, and there was a significant difference, *p* < 0.001 (Figures 5A,D). At the same time, we plotted the progression-free survival (PFS) survival curve of the complete set, and we can see that the progression-free survival rate of the high-risk group was likewise much lower than that of the low-risk group (Figure 5C). After that, the time ROC curves and their AUC values also showed that in the test set AUC: 1 year = 0.679, 3 years = 0.759, 5 years = 0.726; and in the overall set AUC: 1 year = 0.713, 3 years = 0.710, 5 years = 0.760 (Figures 5B,E). Therefore, comparing the AUC values in the training set with the whole set, it can be stated that the risk scoring model still has good performance in predicting long-term prognosis. Furthermore, in the display of risk assessment scatterplots and heatmaps, we also noticed similar outcomes as in the experimental group (Figures 5F–K).

### The clinical value of cuproptosis-related lncRNA risk prediction model

**FIGURE 6:**
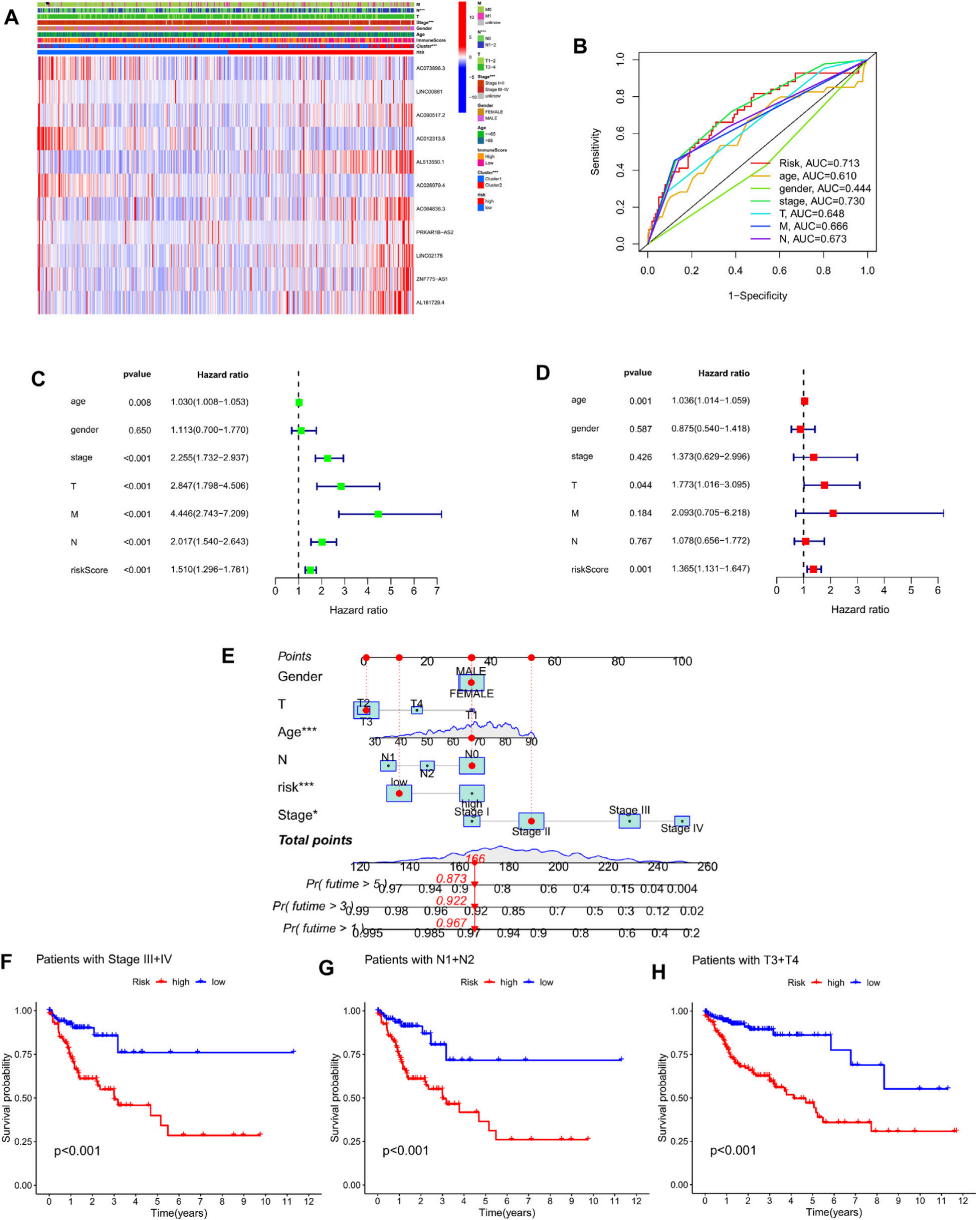
Prognostic clinical value and nomogram plotting of the risk model. **(A)** Heat map showing significant differences in pN stage, clinical stage, and clustering between high-risk and low-risk groups. **(B)** Multivariate ROC curves and AUC values combined with clinicopathological features. **(C–D)** Forest plots of univariate and multivariate Cox regression analysis of variables associated with OS. **(E)** Nomogram for predicting 1-, 3-, and 5-year OS in colon cancer patients. **(F–H)** Survival curves of high- and low-risk groups in StageIII + IV, N1+N2, and T3+T4 patients.

In order to evaluate the clinical value of the Cuproptosis-related lncRNA prediction risk model, first, the clinicopathological characteristics of 446 colorectal cancer patients including age, sex, tumor stage, TNM stage and our previous clustering classification of colorectal cancer tissues were combined, and the association between the expression profiles of 11 copper-dead lncRNAs and the clinical characteristics of low- and high-risk subgroups was shown by heat map (Figure 6A). Secondly, by performing univariate Cox regression analysis and multivariate regression analysis on these characteristics respectively, we can obtain that the two *p* values of the age factor and risk scoring model are both <0.05 and HR values are >1, which proves that the age factor and risk scoring model can be used as Independent prognostic factors in colorectal cancer patients (Figures 6C,E). The multivariate ROC curve of the risk score based on prognostic and clinical variables showed that the AUC of the risk score was 0.713, which was greater than that of age, gender, T stage, N stage, and M stage, and was only lower than the AUC value of stage (Figure 6B). Afterwards, we evaluated the survival results of patients with high and low risk groups in different stages of tumor clinical staging, T staging, and N staging. The Kaplan-Meier survival curve showed that patients with advanced stage had high and medium risk. The survival outcomes of patients in the risk-distributed group were considerably poorer than those in the low-risk group (*p* < 0.001) (Figures 6F–H) (Supplementary Figure S2). Finally, in order to intuitively evaluate the prognosis of colorectal cancer patients, we included the risk scores and clinical variables associated to Cuproptosis into the design of nomograms to predict patients’ 1-year, 3-year, and 5-year OS, The calibration curve proved the reliable predictive usefulness of this nomogram (Figure 6D).

### Principal component analysis and gene set enrichment analysis

**FIGURE 7:**
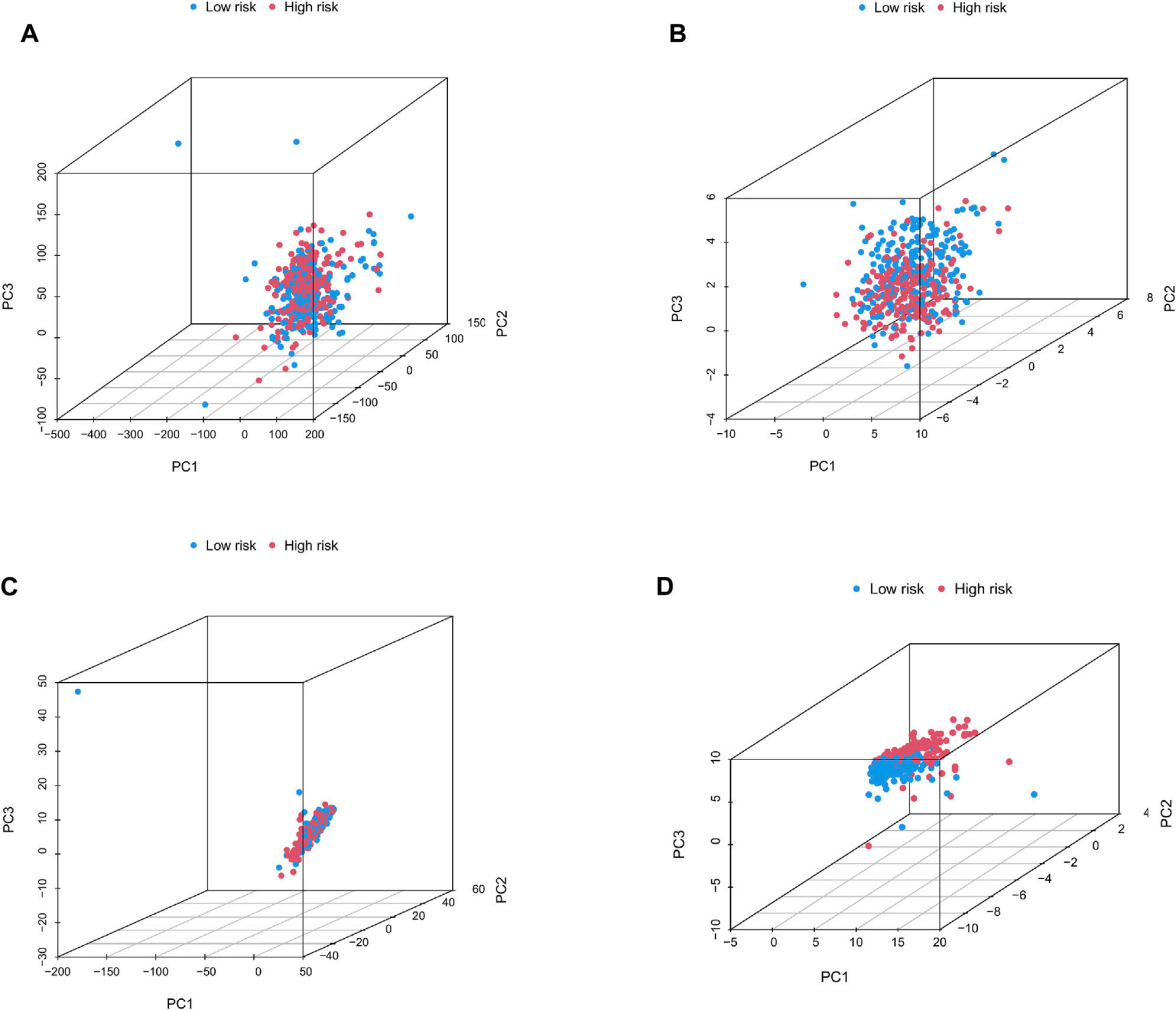
**(A–D)** Principal component distribution map based on the whole transcriptome expression pattern, Cuproptosis-related gene expression pattern, Cuproptosis-related lncRNA expression pattern, and risk scoring model-related lncRNA expression pattern.

**FIGURE 8:**
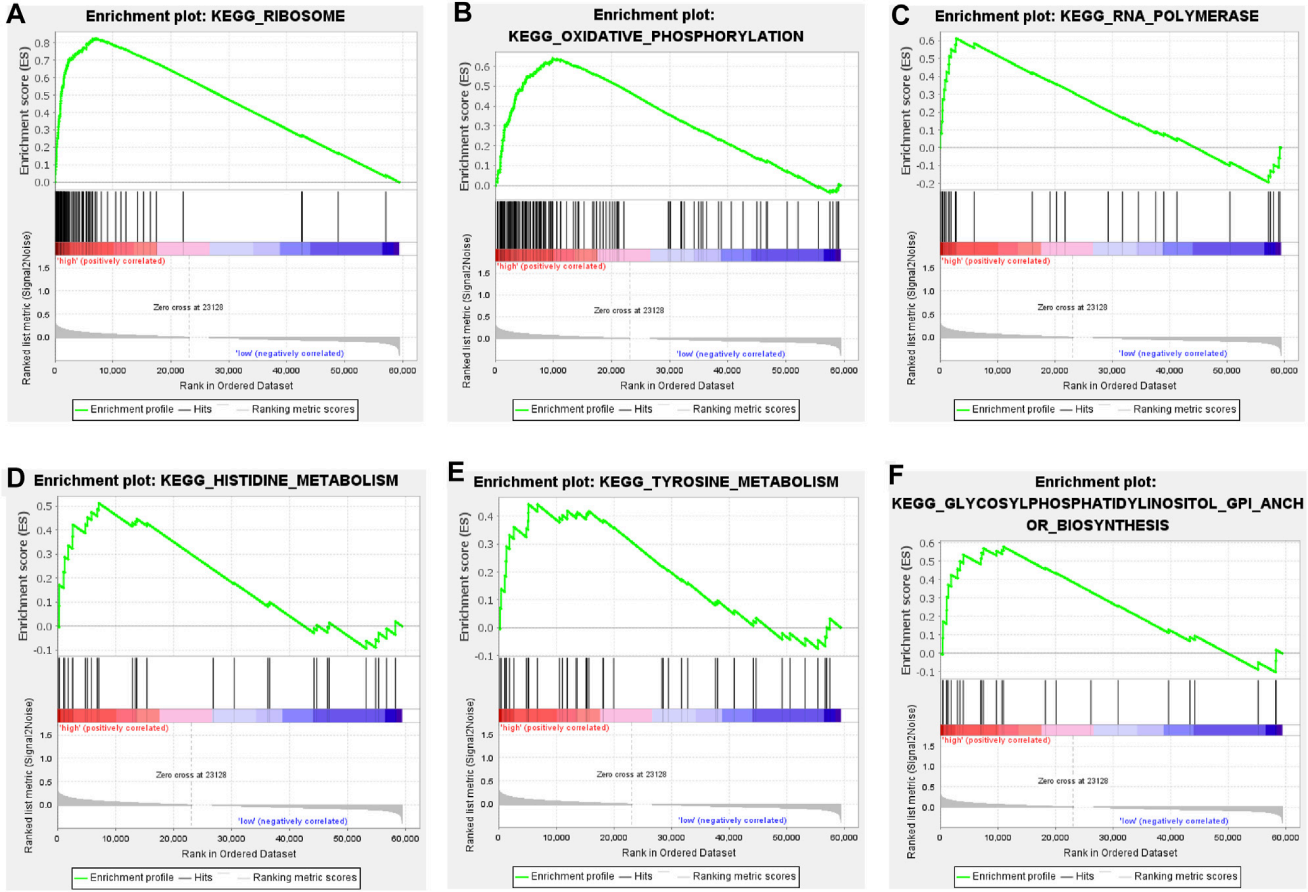
**(A–F)** Pathways significantly enriched in high-risk groups in GSEA KEGG enrichment analysis.

We categorized 446 colorectal cancer patients in the TCGA data set into high and low risk groups according to the Cuprotosis-related lncRNA risk assessment model. PCA analysis was performed based on the entire transcriptome expression pattern (Figure 7A), Cuproptosis-related genes (Figure 7B), Cuproptosis-related lncRNA expression pattern (Figure 7C) and risk score model-related lncRNA expression pattern (Figure 7D), We can see both high and low risk groups had a good regional trends. In order to examine the molecular mechanism and underlying biological processes and pathways of Cuprottosis-related lncRNAs, GSEA was performed between high and low risk groups, and functional annotation was performed. The results showed that the enriched pathways in the high-risk group were predominantly focused on ribosomes, oxidative phosphorylation, RNA polymerase, histidine metabolism, tyrosine metabolism, and glycosylphosphatidylinositol GPI-anchored biosynthesis. The enriched pathways in the low-risk group were clustered in apoptosis, prostate cancer, viral myocarditis, endocytosis, JAK-STAT signaling route, B cell receptor signaling pathway, and ERBB signaling pathway (Figure 8). The detailed findings can be found in the table (Supplementary Tables S1, S2).

### Tumor mutational burden study based on cuproptosis-related lncRNA risk score

**FIGURE 9:**
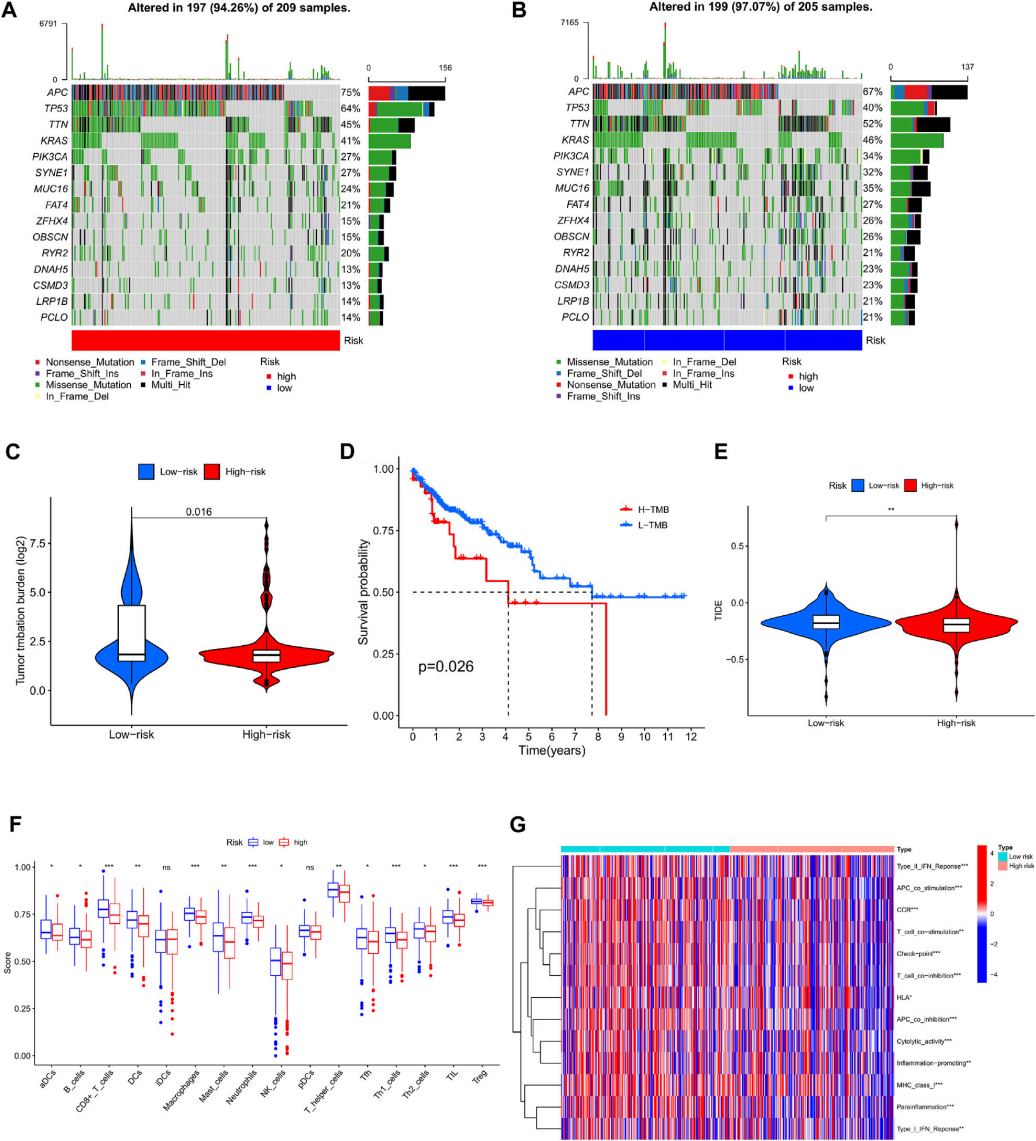
Analysis of tumor mutation burden and immune function in high and low risk groups. **(A)** Waterfall plot of mutant genes in the high-risk group. **(B)** Waterfall plot of mutant genes in the low-risk group. **(C)** Violin plot of TMB contrast in high and low risk groups. **(D)** Kaplan-Meier curves of H-TMB and L-TMB. **(E)** TIDE violin plot between high and low risk groups **(F)** Boxplot comparison of immune infiltrating cells between high and low risk group. **(G)** Comparison of immune-related functions between high and low risk groups.

According to the simple nucleotide variation data downloaded from the TCGA dataset, the tumor mutation burden index of the genes of the patients in the high and low risk groups was calculated respectively, and the TMB in the high risk group was significantly higher than that in the low risk group (*p* = 0.016) (Figure 9C). Comparing the survival results of patients in the high and low TMB groups, the prognosis in the high TMB group was relatively bad (Figure 9D). The top 15 genes with the highest mutation frequency in the mutation spectrum of distinct risk categories are depicted in waterfall plots. In the high-risk group (Figure 9A), mutations were discovered in 197 of 209 samples; in the low-risk group (Figure 9B), mutations were detected in 199 of 205 samples. Longitudinal, mutations in genes such as APC, TP53, TTN, KRAS, PIK3CA, SYNE1, MUC16, FAT4, ZFHX4, OBSCN, RYR2, DNAH5, CSMD3, LRP1B, and PCLO co-occur between high and low groups. Horizontally, among the top 4 genes with high mutation rate, the high-risk group: APC, TP53 mutation frequency is relatively high, and the low-risk group, TTN, KRAS and other mutation frequencies are quite high.

### Analysis of immunological function and immune escape based on risk scoring model

Comparing the infiltration abundance and immune-related functions of immune cells between the high-risk group and the low-risk group (Figure 9F), it can be seen that the infiltration of immune cells in the low-risk group is higher, and Type II IFN Reponse, APC co stimulation, CCR, Tcell co stimulation, Check point, T ell co inhibition, HLA, APC co inhibition, Cytolytic activity, Inflammation promoting, MHC class I, Para inflammation, Type-I IFN Reponse. Expressiveness was low (Figure 9G). Interestingly for the assessment of immune escape potential, the high-risk group had lower TIDE scores than the low-risk group, suggesting that immunotherapy may be relatively more successful in the high-risk group (Figure 9E).

## Discussion

Colorectal cancer is a common high-grade malignant tumor with high morbidity and mortality rates, and according to recent reports, CRC is on the rise in young and middle-aged people (Siegel et al., 2020). With the development of endoscopic surgery for the treatment of precancerous polyps, novel surgical techniques, and the advancement of radiotherapy, chemotherapy and targeted therapy in recent years, the overall survival rate of patients has significantly improved (Lieberman et al., 2000; Chua et al., 2011; Zampino et al., 2016; Ruers et al., 2017). Nevertheless, advanced CRC patients with KRAS mutations and proximal CRC patients frequently have a dismal prognosis (Tsilimigras et al., 2018). Therefore, finding a reliable biomarker that can both predict the prognosis of colorectal cancer patients and identify potentially altered genes in colorectal cancer is important for developing alternative treatment strategies. Tsvetkov et al. recently uncovered cuproptosis, a copper-dependent cell death process that differs from previously reported death processes such as necrosis, apoptosis, autophagy, and ferroptosis, which is a targeted therapy for colorectal cancer. Provides direction. Previous studies have revealed that long non-coding RNA (lncRNA) could influence the growth of colorectal cancer cells *via* gene expression control and activation (Wang et al., 2019). The study by Yang H et al. revealed that lncRNAs could be employed as a therapeutic target for metastatic colorectal cancer (Yang et al., 2020a). However, there has been no research conducted on cuproptosis-related lncRNAs as potential colorectal cancer biomarkers to date.

In this study, we comprehensively analyzed the expression of cuproptosis-related lncRNAs in colorectal cancer, their predictive significance, and their association with tumor mutational load. First, the RNA transcriptome data of 446 colorectal cancer patients were extracted from the TCGA database, and Pearson correlation and Cox regression analysis were performed on 19 cupruptosis-related genes, and 28 prognostic cupruptosis-related lncRNAs were identified. The expression of these lncRNAs in tumor tissues was then found to be different from that in normal tissues. Next, using these differential lncRNAs, we divided colorectal cancer into two subtypes and discovered a significant difference in survival between the two groups. Moreover, the tumor immune microenvironment and potential association with immune targets were investigated in the two distinct clusters. Cluster 2 had a poorer prognosis, a higher immuneScore, stromalScore, and ESTIMATEScore than cluster 1. In addition, PD-L1, CTLA4, HAVCR2, PDCD1, PDCD1LG2, TIGIT and other immune target-related genes were strongly elevated in cluster 1. Meanwhile, correlation analysis revealed that PCED1B-AS1 expression was strongly linked to CTLA4, PDCD1, and TIGIT. These findings are consistent with previous research that suggests immune cell infiltration has a beneficial effect on prognosis (Spector et al., 2019; Wu et al., 2020). According to recent studies, immunotherapy has made comparable progress in cancer treatment (Ohaegbulam et al., 2015), and our findings also suggest that some people with higher immunological ratings may benefit from PD-L1 inhibitor therapy (Zhong et al., 2020; Liu et al., 2021).

Next, LASSO regression and multivariate Cox regression were performed on the lncRNAs with variable prognosis, 11 cuproptosis-related lncRNAs were screened, and the link between these lncRNAs and cuproptosis-related genes was validated through correlation analysis. Among these 11 lncRNAs, Wei J et al. showed that AC073896.3 was associated with autophagy-related genes and impacted the prognosis of colorectal cancer patients (Wei et al., 2020). LINC00861 was identified as a potential immunotherapy intervention target in patients with prostate cancer and was significantly correlated with CTLA4, which is consistent with the findings of our correlation study between prognosis-related lncRNAs and immunological checkpoints (Hu et al., 2021). Elsayed AM et al. demonstrated that PRKAR1B-AS2 could promote tumor growth and confer chemoresistance *via* the PI3K/AKT/mTOR pathway (Elsayed et al., 2021). AL161729.4 was also reported to be related to pyroptosis in bladder cancer (Lu et al., 2022). Based on these 11 lncRNAs, we developed a risk scoring model for colorectal cancer patients using the training set to differentiate between high- and low-risk groups. The survival rate of patients in the high-risk group was lower than that of the low-risk group, and the test set and total set showed consistent results. Importantly, the AUC value of the ROC curve revealed that the scoring model could effectively predict patients’ long-term survival outcomes. Furthermore, multivariate analysis in combination with clinicopathological variables suggested that the model could function as an independent predictor. Meanwhile, the prognosis of patients with different tumor stages was evaluated in the high and low risk groups, revealing that the prognosis of patients with advanced tumors was significantly different between high and low-risk groups. Besides, GSEA demonstrated that these cuproptosis-related lncRNA molecules were significantly enriched in tumor metabolism-related pathways such as histidine metabolism, tyrosine metabolism, oxidative phosphorylation, and other processes that are likely to promote copper induced tumor death mechanisms. Finally, we investigated potentially altered genes in colorectal cancer patients under the influence of cuproptosis-related lncRNAs, and TMB was found to be significantly different between high-risk and low-risk groups. The high TMB group demonstrated a decreased survival rate, while APC, TP53, TTN, and KRAS all revealed high mutation rates in distinct risk groups. Schell MJ and, Yang Y et al. previously revealed the effect of APC, TP53, and KRAS mutations on the prognosis of colorectal cancer patients (Schell et al., 2016; Yang et al., 2020b), and Cen B et al. further found that mutations in the APC gene could lead to resistance of colonic epithelial cells to CD8^+^ T cell cytotoxicity by promoting PD-L1 expression, hence driving tumor immune evasion (Cen et al., 2021). Although studies have shown that KRAS mutation status is unrelated to the effect of anti-PD-1 therapy (Overman et al., 2018), Liao W et al. found that KRAS mutation status in MSS CRC might still be mediated through IRF2 suppression. Furthermore, immune escape may potentially explain the higher TIDE scores in the low-risk group of our study (Liao et al., 2019).

Nonetheless, our study has certain limitations. Firstly, our research data comes from a public database, and, the sample size was small. In addition, there was a shortage of external data sets for additional verification, which could otherwise help further substantiate our data model’s effectiveness. Secondly, we could not decipher the precise mechanism between cuproptosis and immune cell infiltration as well as TMB; thus, it is important to further investigate through basic research.

## Conclusion

In conclusion, we distinguished colorectal cancer molecular subgroups and assessed the immune microenvironment based on 28 cuproptosis-related lncRNAs detected in colorectal cancer samples. A total of 11 lncRNAs were further selected as predictive biomarkers to distinguish between high risk and low-risk groups and identify the differences in immune infiltration and TMB values. Nevertheless, an in-depth understanding of the clinical impact of cuproptosis-related lncRNAs on colorectal cancer progression would better guide treatment regimens and enhance patient outcomes.

## Data Availability

The datasets presented in this study can be found in online repositories. The names of the repository/repositories and accession number(s) can be found in the article/Supplementary Material.

## Supplementary material

Supplementary Figure S1
**(A–D)** Heatmap expression of LAG3, PDCD1, PDCD1LG2, and TIGIT associated with 28 prognostic cuproptosis-related lncRNAs, respectively.

Supplementary Figure S2
**(A–C)** Survival graphs of high and low risk groups in StageI+II, N0, and T1+T2 patients.
