# Supplementary figures and images for: A novel prognostic signature based on cuproptosis-related lncRNA mining in colorectal cancer
Source: Front Genet. 2022 Aug 29;13:969845. doi: 10.3389/fgene.2022.969845 (PMC9465626; doi:10.3389/fgene.2022.969845)

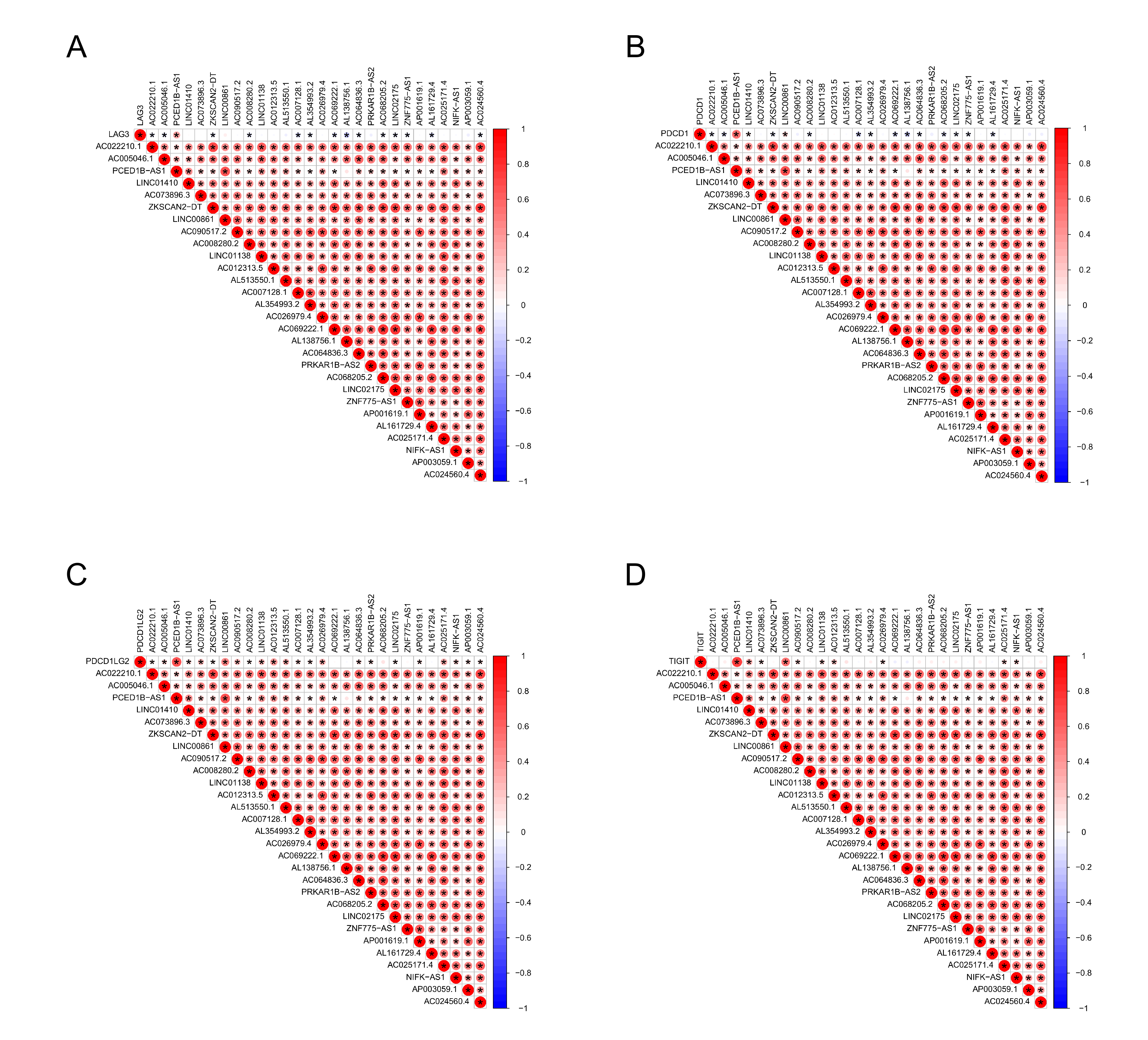

Supplement: Supplementary file 1 [file Image1.tiff]

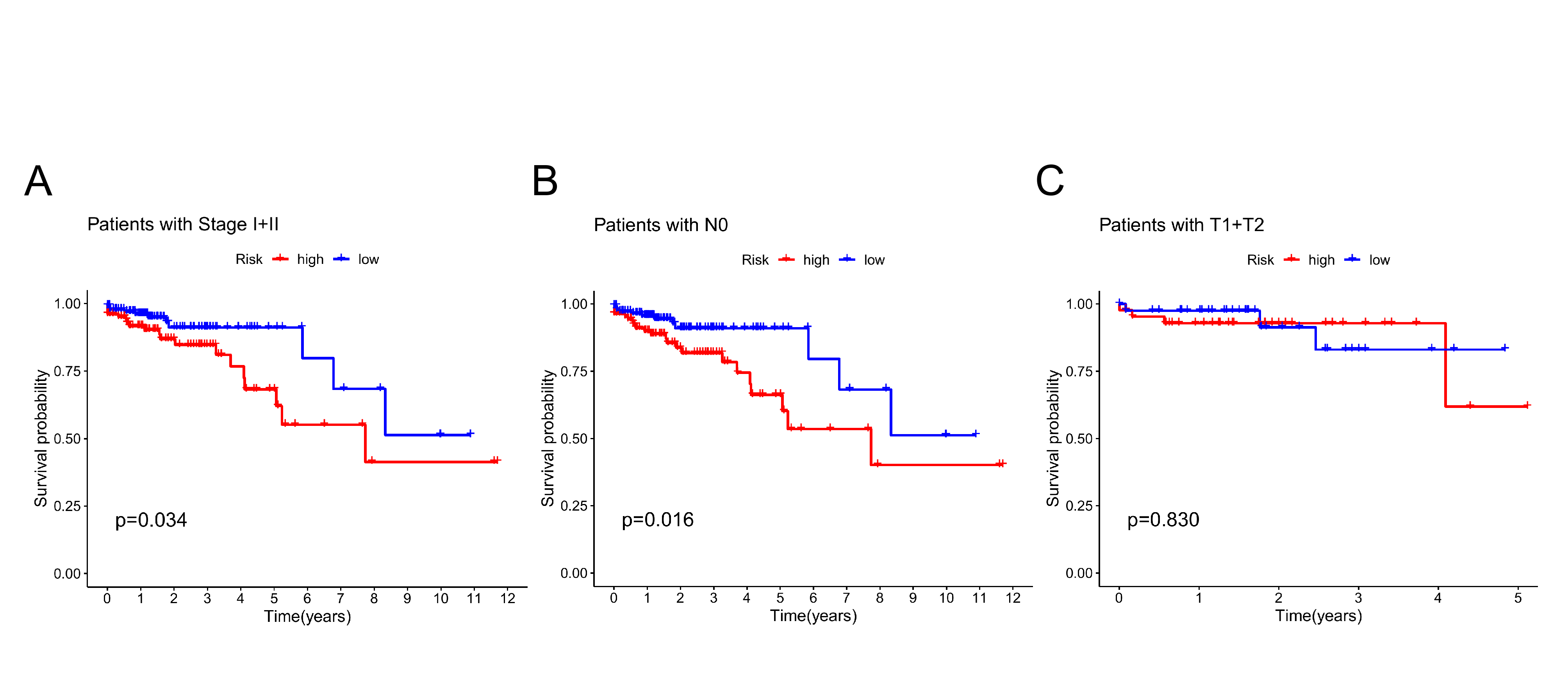

Supplement: Supplementary file 3 [file Image2.tiff]
